# Effects and Safety of a Novel Oral Potassium-Lowering Drug-Sodium Zirconium Cyclosilicate for the Treatment of Hyperkalemia: a Systematic Review and Meta-Analysis

**DOI:** 10.1007/s10557-020-07134-2

**Published:** 2021-01-18

**Authors:** Yaru Zhang, Ruiling Xu, Fanghao Wang, Youxia Liu, Junying Xu, Na Zhao, Fajuan Cheng, Lihong Long, Junya Jia, Shan Lin

**Affiliations:** 1 Department of Nephrology, Hunan Provincial Second People’s Hospital, Hunan, China; 2 Department of Pharmacy, Xiji County People’s Hospital, Ningxia, China; 3 Department of Nephrology, General Hospital of Tianjin Medical University, NO.154, Anshan road, Heping district, Tianjin, China; 4 Department of Nephrology, Qianfoshan Attatched Hospital of Shandong University, Jinan, Shandong province People’s Republic of China; 5 Department of Nephrology, Shandong Provincial Hospital Affiliated to Shandong University, Jinan, Shandong province People’s Republic of China

**Keywords:** Sodium zirconium cyclosilicate (SZC), Hyperkalemia, Serum potassium (sK^+^), Meta-analysis

## Abstract

**Background:**

Oral sodium zirconium cyclosilicate (SZC) is a novel potassium binder capable of achieving a rapid reduction of serum potassium (sK^+^) and maintaining a long-term normokalemia. We undertook a meta-analysis to summarize and evaluate the effects surrounding SZC in patients with hyperkalemia.

**Method:**

We searched data sources from MEDLINE (from 1950 to Sep 2020), EMBASE (from 1970 to Sep 2020), and the Cochrane Library database (from 1950 to Sep 2020) for eligible studies. All randomized controlled trials (RCTs) regarding comparison of therapeutic effects of SZC in hyperkalemia participants were included.

**Results:**

Seven studies, including 1697 patients with hyperkalemia, were analyzed. SZC significantly reduced mean sK^+^ (−0.42 mmol/L; 95% CI: −0.63 to −0.20 mmol/L, *p* = 0.0001) compared with placebo, with a significantly greater proportion of patients with normokalemia (RR 3.48, 95% CI 1.49 to 8.11, *p* = 0.004). Subgroup analyses showed that the longer durations of SZC treatment, the greater magnitudes of potassium reduction when compared with those of placebo (*p* between subgroups = 0.01) at correction phase. Besides, it also demonstrated sK^+^ tended to decrease more in patients who got longer treatment or larger dosage of SZC at maintenance phase; however, the difference did not reach statistical significance. Additionally, the drug was equally effective in studies with larger than 50% of patients with chronic kidney disease (CKD) or diabetes or patients using renin-angiotensin aldosterone system inhibitor (RAAS) inhibitors (all *p* < 0.05). The risk of edema (4.30, 1.17 to 15.84; *p* = 0.03) in SZC group was higher than those of placebo group. No statistically significant differences in the risks of other adverse events were observed between the two groups.

**Conclusions:**

SZC effectively decreased the sK^+^ level in patients with hyperkalemia within 48 h and had benefits in the long-term control of serum potassium in patients who continued to receive SZC with a favorable safety profile from available data.

**Supplementary Information:**

The online version contains supplementary material available at 10.1007/s10557-020-07134-2.

## Introduction

Hyperkalemia is a common electrolyte disorder that can cause potentially life-threatening arrhythmias [1]. An increased risk of hyperkalemia was observed in patients with chronic kidney disease (CKD, 22.8%) [2], heart failure (HF, 13.4%), diabetes (10.8%), and renin-angiotensin-aldosterone system inhibitor (RAASi, 14.2%) use [3–5]. However, the benefit of RAASi treatment in heart and kidney diseases is limited by some side effects, such as increased sK^+^, which is especially severe in patients with renal insufficiency [6]. It is worth noting that after the first hyperkalemia attack, 53.7 or 13.1% of patients experienced RAASi withdrawal or dose reduction, respectively [2]. Some observational cohorts also suggested that hyperkalemia was associated with reduction or cessation of RAAS inhibitors [7–9]. Furthermore, the occurrence of hyperkalemia in CKD patients is closely related to the poor prognosis [2]. Therefore, it is imperative to strengthen the management of hyperkalemia, especially for patients with CKD and hyperkalemia.

Over the years, the treatment for hyperkalemia mainly included low potassium diets, intravenous medications (intravenous insulin + glucose and diuretics), correction of acidosis, and oral medication (such as diuretics, sodium polystyrene sulfonate) [10]. Although intravenous insulin and glucose therapy can quickly reduce serum potassium levels by transferring potassium ions into cells, it does not change the total amount of potassium in the body, and the duration of the efficacy is short and hypoglycemia is a potential complication [10–12]. In addition, diuretics that promote the excretion of potassium ions by the kidneys are not selective for ions, and long-term use can cause renal impairment in patients [11]. Sodium polystyrene sulfonate (SPS) that excrete potassium ions through the intestine also has low selectivity for potassium ions with less compelling evidence from short-term studies (up to a week) for therapeutic effects [10, 13–16]. Furthermore, SPS is also associated with increased serious adverse gastrointestinal events [17].

Sodium zirconium cyclosilicate (SZC) is a stable inorganic crystal, and its structure highly matches the diameter of potassium ions [18, 19]. Its binding force with “potassium” is 25 times than those of other cations [18, 19]. SZC binds potassium ions in the entire digestive tract, which promotes the transfer of sK^+^ to the intestine and eliminates potassium from the body, thereby achieving rapid potassium reduction and long-term management of hyperkalemia [19, 20]. The data showed that the level of sK^+^ decreased significantly after 1 h receiving SZC treatment [21] and 98% of patients returned to normal range within 48 h; nearly 99% of patients had sK^+^ levels < 5.5 mmol/L in the following 1 year [22]. These clinical trials provided evidence on the effectiveness of SZC in reducing sK^+^. Although hundreds of patients were enrolled in each trial, the number of patients in each treatment group was small due to the stratification of drug dosages. Therefore, we conducted this systematic review and meta-analysis by pooling available interventional evidence to better identify the efficacy and safety of SZC in the treatment of hyperkalemia.

## Method

### Data Sources and Searches

Two investigators (Yaru Zhang and Ruiling Xu) independently searched and identified relevant studies from the following data sources: MEDLINE (from 1950 to Sep 2020), EMBASE (from 1970 to Sep 2020), and the Cochrane Library database (from 1950 to Sep 2020) using PubMed and Ovid search engines with the text words of “randomized controlled trial,” “hyperkalemia,” “sodium zirconium cyclosilicate,” or “SZC” or “ZS-9.” Trials were restricted to those published in English language. We also searched relevant reference lists from identified trials and review articles.

### Study Selection

In this meta-analysis, RCTs regarding comparison of therapeutic effects of SZC in patients with hyperkalemia were collected. Inclusion criteria for studies were as follows: (i) study population comprised participants aged 18 years old or older with hyperkalemia (defined as sK^+^ 5.1 mmol/L); (ii) a comparison of SZC and placebo; (iii) reported study outcomes included the change in sK^+^, proportions of responders (defined as patients with sK^+^ < 6.0 mmol/L between 1 and 4 h, and < 5.0 mmol/L at 4 h, and not requiring additional therapy for hyperkalemia, or according to the definition criteria of the included studies), patient with normal sK^+^concentration (defined as patients with sK^+^ < 5.1 mmol/L, or according to the definition criteria of the included studies) between the SZC and placebo group at the end of study, and/or safety data of drug-related adverse events; and (iv) study design was RCT and sample size was larger than 20. We excluded studies without control group or lack of available data.

### Data Extraction and Quality Assessment

We extracted information using standard data extraction forms, which included baseline patient characteristics, intervention, doses of drug, follow-up duration, outcome events, and adverse events. We used standard criteria (Cochrane risk of bias tool) to assess the inherent risk of bias of trials, as showed in Table S1. Two investigators (Yaru Zhang and Ruiling Xu) independently undertook data extraction and quality assessment using a standardized approach. Any disagreements between two investigators were resolved by consultation with a third reviewer (Youxia Liu). The study was conducted according to the PRISMA guidelines.

### Data Synthesis and Statistical Analyses

We calculated relative risk (RR) and 95% confidence interval (CI) for outcome of proportions of responders with normokalemia using random-effects model. Weighted mean difference and standard deviation (SD) between groups were applied for continuous variable of the change in sK^+^. We analyzed heterogeneity by I^2^ statistic to describe the percentage of variability. Meta-regression was conducted to evaluate the impact of different dosages of SZC on the change of sK^+^. Begg Funnel plot was performed to assess potential publication bias. The results were considered significant with 2-sided *p* < 0.05. STATA, version 12.0 and Review Manager 5.1 software, were used to perform this meta-analysis. For data with high heterogeneity, we performed sensitivity analysis or subgroup analysis to check for instability and change in significance of the effect estimate.

## Results

### Study Characteristics and Quality Assessment

**Fig. 1:**
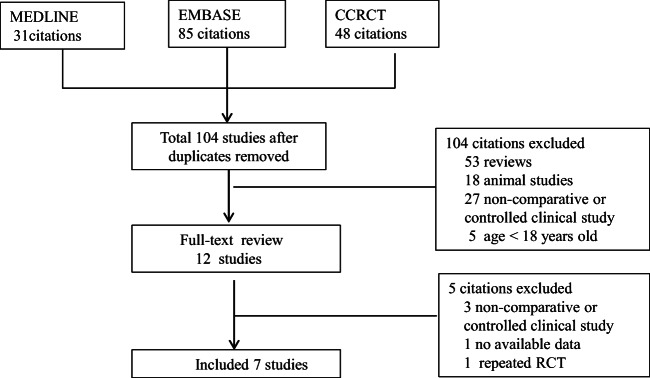
Process for identifying studies eligible for the meta-analysis

The literature search yielded 104 articles, and eventually, seven studies with 1697 patients were included in our meta-analysis according to the inclusion criteria (Fig. 1). Of the contained seven trials, all of them compared the efficacy of SZC with placebo. These studies were performed from 2014 to 2020, with sample sizes ranging from 70 to 754 and treatment duration ranging from 4 h to 28 days (Table 1). Baseline characteristics of patients in trials were estimated, and the results showed that the mean age was 65 ± 13 years, the mean serum potassium was 5.3 ± 0.8 mmol/L, and the mean weight was 83.7 ± 22.4 kg.

**Table 1:** Characteristics of the randomized controlled studies included in meta-analysis for Sodium Zirconium Cyclosilicate (SZC)

Study	Baseline sK+(mmol/L)	Number of patients	Treatment		Duration	Endpoint results	Medical history: n (%)	RAASi: n (%)
			SZC	control				
David 2015 Phase 3	5.36 ± 0.66	I-phasen =754	I-phase: SZC 1.25-10 g tid	I-phase: placebo	I-phase: 48 h	Change in sK+ at 48 h	CKD: 463 (61.5%); HF: 300 (39.8%); DM: 451 (59.9%)	502 (66.7%)
		M-phase *n* = 543	M-phase: SZC 1.25-10 g tid	M-phase: placebo	M-phase:14 days			
DIALIZE 2019 Phase 3b	NA	*n* = 196	SZC 5 g qd or placebo on nondialysis days	placebo	4 weeks	Proportion of responders	CKD: 196 (100%)	NA
ENERGIZE 2020 Phase 2	6.43 ± 0.7	*n* = 70	10 g SZC tid + insulin + glucose during a 10-h period	placebo + insulin + glucose	4 h	Change in sK+ at 4 h	CKD: 25 (41.7%), HF: NA; DM: 21(30%)	25 (35.7%)
HARMONIZE 2014 Phase 3	5.6 ± 0.4	I-phase *n* = 258	I-phase: SZC 10 g tid		I-phase: 48 h	Proportion of patient with normokalemia	CKD: 152 (64.1%); HF: 87 (36.7%); DM: 157 (66.2%)	163 (68.8%)
		M-phase *n* = 237	M-phase: SZC 5-15 g/day	M-phase: placebo	M-phase:14 days			
HARMONIZE 2019 Phase 3	5.71 ± 0.5	I-phase *n* = 267	I-phase: SZC 10 g tid for 48 h		I-phase: 48 h	Proportion of patient with normokalemia	CKD: 209783%); HF: 50 (18.7%); DM: 172 (64.4%)	204 (76.4%)
		M-phase *n* = 248	M-phase: SZC 5 g and 10 g qd for 28 days	M-phase: placebo	M-phase:28 days			
Kashihara2020 Phase 2/3	5.6 ± 0.4	103	SZC 5 g and10g tid for 48 h	placebo	48 h	Proportion of patient with normokalemia	CKD: 78 (75.5%); HF: 14 (13.61%); DM: 62 (60.2%)	80 (77.7%)
Stephen 2015 Phase 2	5.09 ± 0.37	*n* = 90	SZC 0.3 g, 3 g, and 10 g tid	placebo	48 h	Change in sK+ at 48 h	CKD: 56(60.9%); HF: NA; DM: 50(55.5%)	NA

SZC, Sodium zirconium cyclosilicate; I-phase, initial phase(or correction phase); M-phase, maintenance phase; tid, three times daily; qd, once daily; CKD, Chronic kidney disease; DM, Diabetic mellitus; HF, Heart failure; RAASi, renin-angiotensinaldosterone system inhibitor; NA, not available

The inherent risk of bias of trials was performed for all studies by Cochrane risk of bias tool (including sequence generation, allocation concealment, blinding, incomplete outcome data, selective outcome reporting, and other source of bias). As listed in Table S1, all studies had generally low risk of bias in all items.

### Treatment Outcomes

**Fig. 2:**
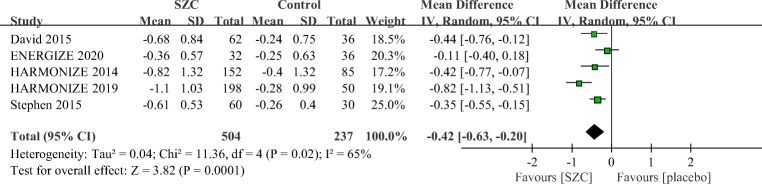
Comparison of change in serum potassium (sK^+^) between the SZC and placebo group. SZC, Sodium zirconium cyclosilicate

**Fig. 3:**
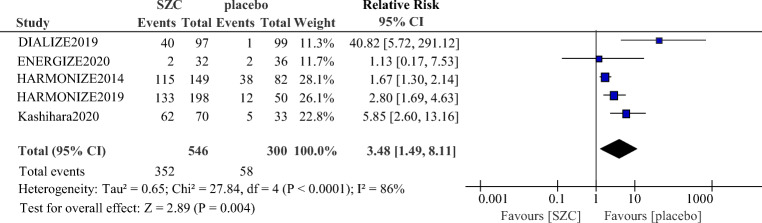
Comparison of proportions of responders with normokalemia between the SZC and placebo group. SZC, Sodium zirconium cyclosilicate

As showed in Fig. 2, patients who received SZC had a significant reduction of sK^+^ (−0.42 mmol/L; 95% CI: −0.63 to −0.20 mmol/L, *p* = 0.0001) compared with those who received placebo. SZC also significantly increased the proportion of responders with normokalemia (RR 3.48, 95% CI 1.49 to 8.11, *p* = 0.004, Fig. 3).

**Fig. 4:**
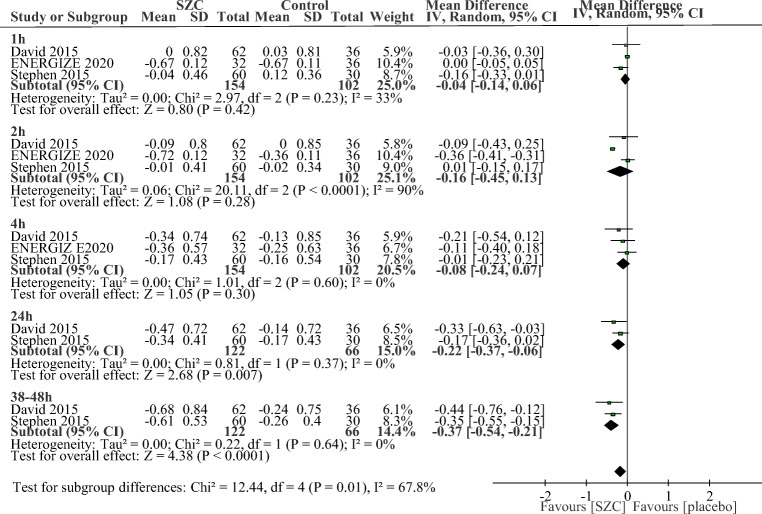
Time-related subgroup analysis of change in serum potassium (sK^+^) between SZC and placebo group at correction phase (48 h) after start of treatment. SZC, Sodium zirconium cyclosilicate

**Fig. 5:**
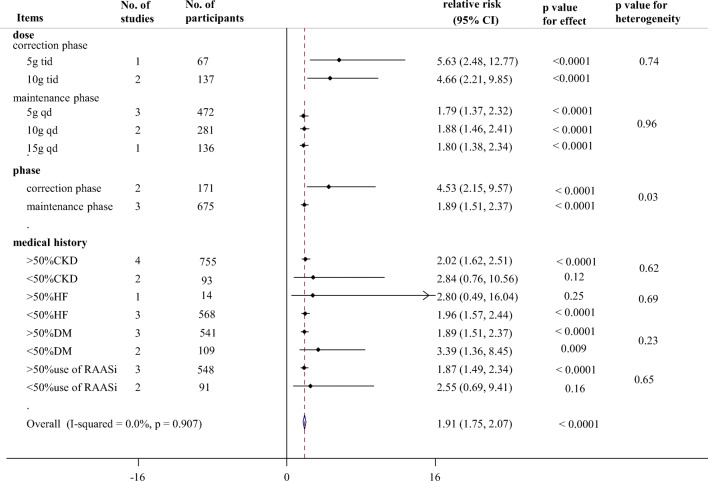
Summary of subgroup analyses for proportions of responders with normokalemia between the SZC and placebo group. >50% CKD: the proportion of patients with CKD was larger than 50% in the included studies; SZC, Sodium zirconium cyclosilicate; CKD, chronic kidney disease; DM, diabetic mellitus; RAASi, renin-angiotensin aldosterone system inhibitor; tid: three times daily; qd: once daily; HF, heart failure

The drug was equally effective across various subgroups, including treatment duration, phase, dosage, and percentage of patients with CKD or diabetes or patients using RAAS inhibitor. We found that the longer durations of SZC treatment, the greater magnitudes of potassium reduction when compared with those of placebo at correction phase (p between subgroups = 0.01, Fig. 4). As shown in Fig. 4, greater reductions in sK^+^ were achieved by SZC at 24 h (−0.22 mmol/L; −0.37 to −0.06 mmol/L, *p* = 0.007) and 38–48 h (−0.37 mmol/L; −0.54 to −0.21 mmol/L, *p* < 0.0001) compared with placebo. However, SZC did not show significant advantages in the decrease of sK^+^ at 1 h (−0.04 mmol/L; −0.14 to 0.06 mmol/L), 2 h (−0.16 mmol/L; −0.45 to 0.13 mmol/L, *p* = 0.28) and 4 h (−0.08 mmol/L; −0.24 to 0.07 mmol/L, *p* = 0.30) when compared with placebo. At maintenance phase, SZC treatment were also associated with significant potassium reductions on day 15 (−0.47 mmol/L; −0.74 to −0.20 mmol/L, *p* = 0.0006, Supplement Fig. 1) and day 29 (−0.75 mmol/L; −0.98 to −0.51 mmol/L, *p* < 0.0001, Supplement Fig. 1). Besides, the proportions of responders with normokalemia were higher in patients with SZC, with RRs of 4.53 (95% CI 2.15 to 9.57, *p* < 0.0001, Fig. 5) at correction phase and 1.89 (95% CI 1.51 to 2.37, *p* < 0.0001, Fig. 5) at maintenance phase, and the RR at correction phase was significantly higher than that at maintenance phase (*p* between the two phase = 0.03, Fig. 5).

**Fig. 6:**
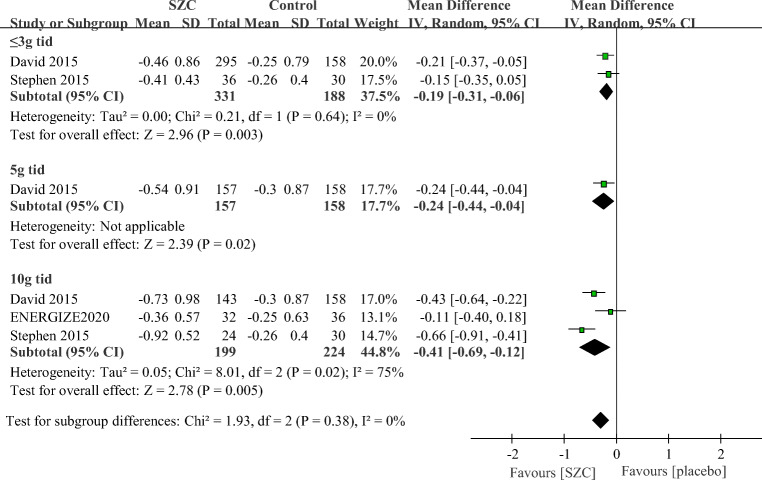
Subgroup analysis of different doses in the change of serum potassium (sK^+^) between the ZS-9 and placebo group at correction phase (48 h) after start of treatment. SZC, Sodium zirconium cyclosilicate; tid: three times daily

Subgroup analyses showed that patients taking SZC 10 g orally three times a day (tid) significantly decreased sK^+^ level to the greatest extent (−0.41 mmol/L; −0.69 to −0.12 mmol/L, *p* = 0.005, Fig. 6), followed by 5 g tid (−0.24 mmol/L; −0.44 to −0.04 mmol/L, *p* = 0.02) and 3 g tid (−0.19 mmol/L; −0.31 to −0.06 mmol/L, *p* = 0.003) compared with those who received placebo at correction phase. At maintenance phase, three different doses (5 g qd, 10 g qd and 15 g qd) of SZC could significantly decrease the levels of sK^+^ (−0.53 mmol/L; −0.79 to −0.27 mmol/L, p < 0.0001; −0.92 mmol/L; −1.25 to −0.59 mmol/L, p < 0.0001; −0.80 mmol/L; −1.27 to −0.33 mmol/L, *p* = 0.0009, Supplement Fig. 2), respectively. Meta-regression analyses showed that the different dosages of SZC had no significant effect on the change of sK^+^ at correction phase (*p* = 0.61) and maintenance phase (*p* = 0.17). For the proportion of responders with normokalemia (Fig. 5), the results also showed the RRs were higher in patients who received SZC (5.63, 2.48–12.77; 4.66, 2.21–9.85 for 5 g tid and 10 g tid doses at correction phase; 1.79, 1.37–2.32; 1.88, 1.46–2.41; 1.80, 1.38–2.34 for 5 g qd, 10 g qd, and 15 g doses, respectively, at maintenance phase).

**Fig. 7:**
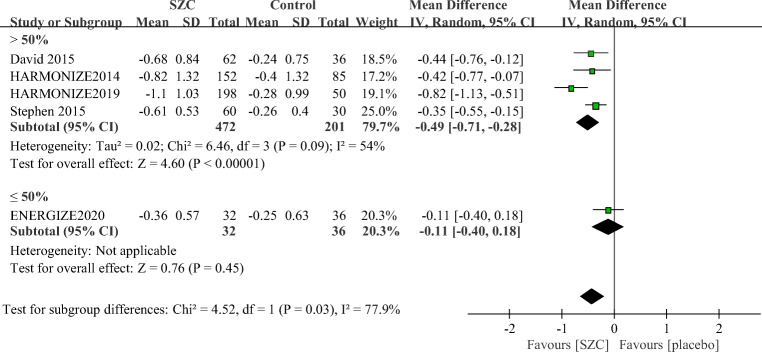
Subgroup analysis according to the proportion of patients with CKD or DM or patients using RAAS inhibitors in the included studies of change in serum potassium (sK^+^) between the ZS-9 group and placebo group. SZC, Sodium zirconium cyclosilicate; CKD, chronic kidney disease; DM, diabetic mellitus; RAASi, renin-angiotensin aldosterone system inhibitor

Additionally, as shown in Fig. 5, a higher proportion of patients in SZC group attained normokalemia at the end of their treatment compared with those in the placebo group in studies with more than 50% of patients with CKD (2.08, 1.71–2.53), diabetes (1.96, 1.60–2.40), or RAASi use (1.94, 1.58–2.38). There was no significant difference in proportions of responders with normokalemia between the SZC and placebo group in studies with more than 50% of patients with HF (2.80, 0.49–16.04, *p* = 25). Besides, there were no significant differences between subgroups based on the proportion of those patients above and below 50%. Similarly, a greater reduction in sK^+^ was achieved with SZC compared with placebo in studies with more than 50% of patients with CKD, HF, diabetes, or patients using RAAS inhibitors (−0.49 mmol/L; −0.71 to −0.28 mmol/L, *p* < 0.0001, Fig. 7).

### Adverse Effects

Table 2 displayed the pooled clinical trial results of adverse events. Among adverse events of SZC, the risk of edema in SZC group was 4.30 times higher than that of placebo group (4.30, 1.17 to 15.84; *p* = 0.03). No statistically significant differences were observed in the risks of other adverse events including any adverse event, serious adverse event, gastrointestinal disorders (including nausea, vomiting, diarrhea, and constipation), cardiac disorders, urinary tract infection, hypokalemia between SZC, and placebo therapy (Table 2, all *p* > 0.05).

**Table 2:** Comparison of adverse events between the SZC and placebo group

Adverse events		Reporting Study (n)	SZC group (n/n)	Placebo group (n/n)	RR (95%CI)	*p* value
Any Adverse Event		7	289/1205	116/492	1.25(0.98,1.60)	0.07
Serious Adverse Event		4	27/480	14/271	1.42 (0.71, 2.84)	0.33
Gastrointestinal disorders	Nausea	4	12/749	4/251	1.49(0.51, 4.42)	0.47
	Diarrhea	4	20/950	11/337	0.77(0.36, 1.65)	0.5
	vomiting	2	4/655	3/188	0.47(0.04, 5.04)	0.53
	Constipation	4	17/517	9/267	0.91(0.22, 3.82)	0.9
Cardiac disorders		3	18/945	2/293	1.72 (0.51, 576)	0.38
Urinary tract infection		3	8/853	0/238	2.19 (0.39, 12.30)	0.37
Edema		2	32/350	2/135	4.30 (1.17, 15.84)	**0.03**
Hypokalemia		3	6/383	3/172	1.42 (0.39, 5.24)	0.6

RR: Relative Risk; SZC: Sodium zirconium cyclosilicate

#### Sensitivity Analysis and Publication Bias

Sensitivity analysis was performed for change in sK^+^ and the proportion of responders with normokalemia. The results revealed that no studies had a significant effect on the results as showed in Supplement Fig. 3. Statistical testing showed no evidence of publication bias for the change of the proportion of responders with normokalemia, which was displayed in Supplement Fig. 4.

## Discussion

This systematic review and meta-analysis was conducted to estimate the efficacy and safety of SZC for hyperkalemia, and eventually, 8 RCTs met our inclusion criteria [21, 23–29]. Our results showed that SZC effectively decreased the sK^+^ in patients with hyperkalemia within 48 h and had benefits for the long-term control of sK^+^ in patients who continued to receive SZC with a favorable safety profile from available data. Compared with placebo, the advantages in decreasing sK^+^ of SZC at 1, 2, and 4 h were not found according to our results. Besides, SZC seemed to safely and effectively normalize and maintain potassium levels in patients with CKD, HF, diabetes, or RAASi use.

Consistent with our findings, available data showed that SZC was associated with a significant decrease in sK^+^ within 48 h and had benefits for the long-term control of sK^+^. A previous meta-analyses and systematic review published in 2017 evaluated the effects of patiromer and SZC on hyperkalemia, and the results showed that SZC reduced sK^+^ 0.67 mEq/L at 48 h [30]. Kosiborod et al. [24] found that 84 and 98% of patients had normal sK^+^ concentration by 24 h and 48 h with SZC treatment, respectively. Several published studies evaluated the long-term effects of potassium control. Bruce et al. [22] found most participants (82%) achieved normokalemia after administration of SZC 30 g (three 10-g doses) over 24 h and the treatment with SZC resulted in a reduction in potassium from ≥ 5.1 mmol/L at baseline to 4.7 mmol/L within 3–12 months. In HARMONIZE study with an 11-month open-label extension, 88.3% of patients maintained mean sK^+^ within the normokalemic range for ≤ 11 months during ongoing SZC treatment [31]. In our study, SZC was associated with a decrease in sK^+^ levels and a higher proportion of patients with normokalemia with 48 h and maintained normokalemia during 2–4 weeks. The present study provided additional evidence to support the viewpoint that SZC contributed to effectively reducing sK^+^ concentration and maintaining long-term normokalemia. However, compared with placebo, the effects for decreasing sK^+^ of SZC at 1, 2, and 4 h were not found according to our study. A study conducted by Mikhail showed that in population with mean sK^+^ level at baseline larger than 6.0 mmol per liter, the mean sK^+^ level declined by 0.4 mmol per liter (95% CI, 0.2 to 0.5) at 1 h, by 0.6 mmol per liter (95% CI, 0.4 to 0.8) at 2 h, and by 0.7 mmol per liter (95% CI, 0.6–0.9) at 4 h (*p* < 0.001 for the comparison of each time point with baseline) after one 10-g dose of SZC [32]. A study published in The New England journal of medicine showed that the SZC significantly decreased sK^+^ from baseline by 0.11 mEq/l at 1 h after the first 10-g dose, as compared with an increase of 0.01 mmol per liter in the placebo group (*p* = 0.009). This disparity may be contributed by that the dose of SZC in the above studies were 10 g, and the dose of SZC in our study included 0.3 g, 3 g, etc., which may have weakened the effect of SZC in reducing sK^+^. We did not conduct a subgroup analysis of the potassium-lowering effect of different doses of SZC within 4 h because of poorly available data. The study conducted by Naoki [29] showed that median time to normalization of sK^+^ concentration was shorter with SZC 10 g versus placebo (1.8 h vs. 3.9 h) and was similar for the SZC 5 g and placebo groups (3.9 and 3.9 h, respectively), suggesting that the time to normalization of sK^+^ concentration was shorter with larger dose of SZC. Thus the absence of positive effects at 1, 2, and 4 h in our meta-analysis should not be considered as conflictive with the general literature. We believe that when more and more large-scale studies are included in the future, the advantages of SZC in rapidly reducing the sK^+^ of patients with hyperkalemia, especially those with severe hyperkalemia, will be more prominent.

Our further subgroup analysis revealed that compared with placebo, three doses of SZC at correction phase (10 g tid, 5 g tid, or ≤ 3 g tid) and at maintenance phase (15 g qd, 10 g qd, or 5 g qd) resulted in the decrease of sK^+^ levels and a higher proportion of patients with normokalemia. It demonstrated patients with hyperkalemia got larger dosage of SZC their sK^+^ tended to decrease more; however, the difference did not reach statistical significance. The study conducted by Naoki [29] showed that the exponential rates of sK^+^ change from 0 to 48 h versus placebo were greater with larger doses of SZC, and they also found the time for sK^+^ to return to normal level was shorter with larger dose of SZC, suggesting that SZC lowed sK^+^ with dose dependency.

As mentioned above, CKD, HF, diabetes, or the use of RAASi were high risks for hyperkalemia [2]. Kosiborod et al. found SZC reduced and maintained normal potassium for up to 4 weeks in patients with CKD, heart failure, and diabetes [24]. A study of hyperkalemia patients with renal insufficiency found that during SZC treatment, the average sK^+^ levels decreased from 5.7 to 4.8 mmol/L in patients with eGFR < 30 mL/min/1.73 m2 and from 5.6 to 4.7 mmol/L in those with eGFR ≥30 mL/min/1.73 m2 at day 365 [33]. They also pointed the proportions of patients who reached normal potassium during the maintenance period were 82 and 90% for the eGFR < 30 and ≥ 30 mL/min/1.73 m2 group at day 365, respectively. In a recent Dialize study, the use of SZC in patients undergoing hemodialysis was able to maintain sK^+^ levels between 4.0 and 5.0 mmol/L during dialysis with a few records of adverse events [27]. Hyperkalemia usually limits the use of RAASi in patients who were expected to derive the most benefit from these classes of drugs. Bruce et al. [22] found that 87% of patients continued to take RAASi or increased their dose among 483 RAASi users with hyperkalemia at baseline and 14% started RAASi treatment among 263 RAASi-naïve participants after initiating SZC. A study conducted by Jared et al. [34] demonstrated that SZC safely and effectively normalized and maintained potassium levels (71–85% vs 48% for placebo, *p* < 0.01) and prevented hyperkalemia from redeveloping in patients receiving RAASi for up to 4 and 8 weeks. These results were generally in accordance with our results, suggesting that SZC was a potentially effective treatment for patients with CKD or diabetes or those receiving RAASi treatment. However, there was no significant difference in proportions of responders with normokalemia between the SZC and placebo group in studies with more than 50% of patients with HF according to our meta-analysis. We further analyzed the reasons and found that this result was driven by only one study with a small sample size. Therefore, the identification of sub-groups of patients who may benefit is important to help guide treatment and should be explored in future studies.

The safety of SZC is the main issues of concern. The current research reported the side effects of SZC mainly included gastrointestinal disorders (including nausea, vomiting, diarrhea and constipation), cardiac disorders, urinary tract infection, edema and hypokalemia [20–30, 33]. In our meta-analysis, of all adverse events mentioned above, SZC only increased the risk of edema compared with placebo. No statistically significant differences in the risks of other adverse events including any adverse event, serious adverse event, gastrointestinal disorders (including nausea, vomiting, diarrhea, and constipation), cardiac disorders, urinary tract infection and hypokalemia were found between SZC and placebo therapy. These results indicated that SZC had a favorable safety.

A larger sample size and rigorous statistic methodology were the strengths of this meta-analysis. The overall low risk of bias of included studies was an additional strength of this review. However, our study had some limitations. Firstly, scant primary data prevented us from undertaking subgroup analysis of effect of SZC in patients with different degrees of hyperkalemia, especially in patients with severe hyperkalemia. Secondly, the duration of drug treatment and follow-up of the included studies in our study did not exceed 2 months. The safety is still uncertain when SZC is used for more than 1 year. Thirdly, we did not register this analysis with PROSPERO. More and larger RCTs with longer maintenance-period are needed in the future. Fourthly, in the meta-analysis, studies with open-label extension were included, which may introduce a bias where patients who do not tolerate the drug are unlikely to take part in the extension study.

## Conclusion

SZC effectively decreased the sK^+^ in patients with hyperkalemia within 48 h and had benefits in the long-term control of sK^+^ in patients who continued to receive SZC with a favorable safety profile from available data. Besides, SZC seemed to safely and effectively normalize and maintain potassium levels in patients with CKD or diabetes or those receiving RAASi treatment. We believe that when more and more large-scale studies are included in the future, the advantages of SZC in rapidly reducing the sK^+^ of patients with hyperkalemia, especially those with severe hyperkalemia, will be more prominent.

## Supplementary Information

ESM 1 (PDF 229 kb)

ESM 2 (DOCX 30 kb)

## Data Availability

Not applicable.
