# Supplementary material for: Effects and Safety of a Novel Oral Potassium-Lowering Drug-Sodium Zirconium Cyclosilicate for the Treatment of Hyperkalemia: a Systematic Review and Meta-Analysis
Source: Cardiovasc Drugs Ther. 2021 Jan 18;35(5):1057–66. doi: 10.1007/s10557-020-07134-2 (PMC8452568; doi:10.1007/s10557-020-07134-2)
Supplement: Supplementary file 1 — (PDF 229 kb) [file 10557_2020_7134_MOESM1_ESM.pdf]

	SZC			Control				Mean Difference
**Study or Subgroup**	**Mean**	**SD**	**Total**	**Mean**	**SD**	**Total**	**Weight**	**IV, Random, 95% CI**
**Day 15**								
David 2015	-0.71	1.26	62	-0.33	0.85	36	17.8%	-0.38 [-0.80, 0.04]
HARMONIZE2014	-1.04	1.26	143	-0.5	1.3	80	25.1%	-0.54 [-0.89, -0.19]
**Subtotal (95% CI)**			**205**			**116**	**42.9%**	**-0.47 [-0.74, -0.20]**
Heterogeneity: $Tau^2 = 0.00$; $Chi^2 = 0.33$, df = 1 (P = 0.57); $I^2 = 0\%$								
Test for overall effect: Z = 3.45 (P = 0.0006)								
**Day 29**								
HARMONIZE2014	-1.05	1.29	149	-0.4	1.32	82	24.9%	-0.65 [-1.00, -0.30]
HARMONIZE2019	-1.1	1.03	198	-0.28	0.99	50	32.1%	-0.82 [-1.13, -0.51]
**Subtotal (95% CI)**			**347**			**132**	**57.1%**	**-0.75 [-0.98, -0.51]**
Heterogeneity: $Tau^2 = 0.00$; $Chi^2 = 0.50$, df = 1 (P = 0.48); $I^2 = 0\%$								
Test for overall effect: Z = 6.28 (P < 0.00001)								

Test for subgroup differences: $Chi^2 = 2.25$, df = 1 (P = 0.13), $I^2 = 55.5\%$

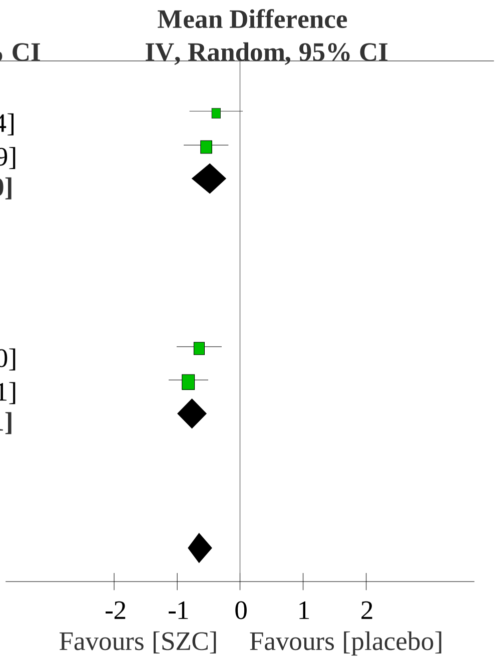

Supplement Figure 1. Time-related subgroup analysis of change in serum potassium (sK^+) between SZC and placebo group at maintenance phase. SZC, Sodium zirconium cyclosilicate.

Study or Subgroup	SZC Mean	SZC SD	SZC Total	Control Mean	Control SD	Control Total	Weight	Mean Difference IV, Random, 95% CI	Mean Difference IV, Random, 95% CI
**5g qd**									
HARMONIZE2014	-0.8	0.98	45	-0.4	1.32	82	19.7%	-0.40 [-0.80, 0.00]	
HARMONIZE2019	-0.9	1.05	99	-0.28	0.99	50	23.8%	-0.62 [-0.96, -0.28]	
**Subtotal (95% CI)**			**144**			**132**	**43.5%**	**-0.53 [-0.79, -0.27]**	
Heterogeneity: Tau² = 0.00; Chi² = 0.66, df = 1 (P = 0.42); I² = 0%									
Test for overall effect: Z = 3.95 (P < 0.0001)									
**10g qd**									
HARMONIZE2014	-1.1	1.37	50	-0.4	1.32	82	16.0%	-0.70 [-1.18, -0.22]	
HARMONIZE2019	-1.33	0.96	99	-0.28	0.99	50	24.6%	-1.05 [-1.38, -0.72]	
**Subtotal (95% CI)**			**149**			**132**	**40.5%**	**-0.92 [-1.25, -0.59]**	
Heterogeneity: Tau² = 0.02; Chi² = 1.40, df = 1 (P = 0.24); I² = 28%									
Test for overall effect: Z = 5.41 (P < 0.00001)									
**15g qd**									
HARMONIZE2014	-1.2	1.42	54	-0.4	1.32	82	16.0%	-0.80 [-1.27, -0.33]	
**Subtotal (95% CI)**			**54**			**82**	**16.0%**	**-0.80 [-1.27, -0.33]**	
Heterogeneity: Not applicable									
Test for overall effect: Z = 3.31 (P = 0.0009)									

Test for subgroup differences: Chi² = 3.48, df = 2 (P = 0.18), I² = 42.5%

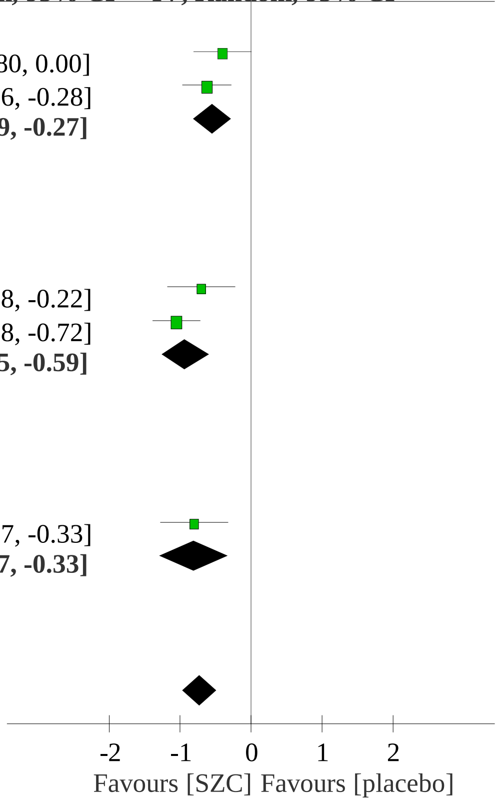

Supplement Figure 2. Subgroup analysis of different doses in the change of serum potassium (sK^+) between the SZC and placebo group at maintenance phase; SZC, Sodium zirconium cyclosilicate; qd: once daily.

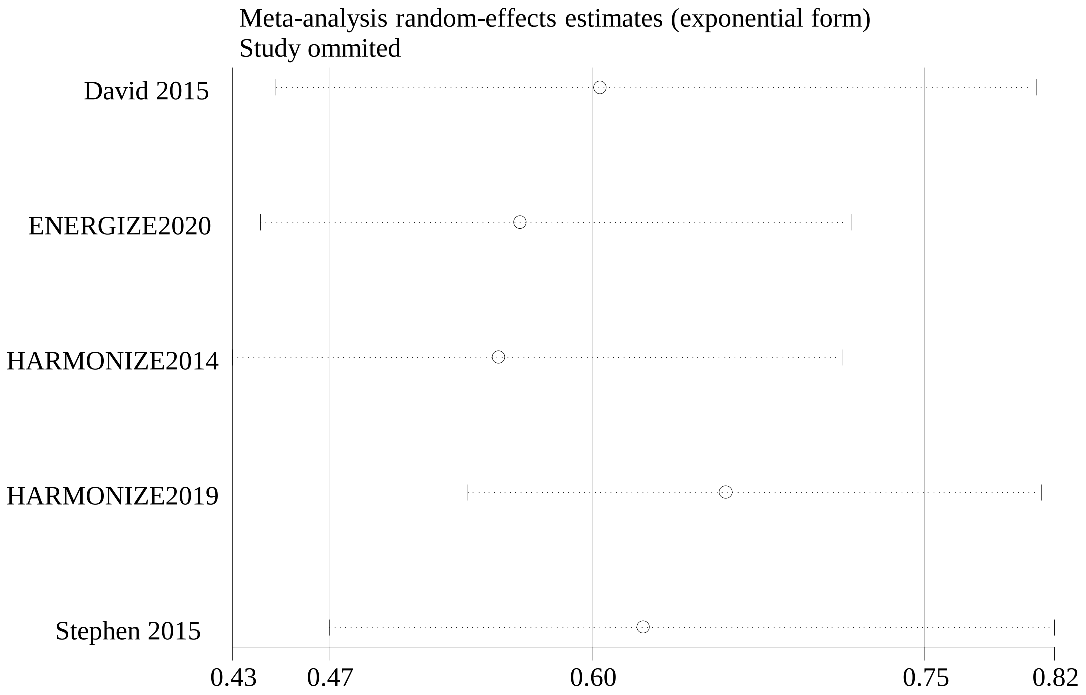

Supplement Figure 3. Sensitivity analysis for change in serum potassium (sK^+) between the SZC and placebo group.

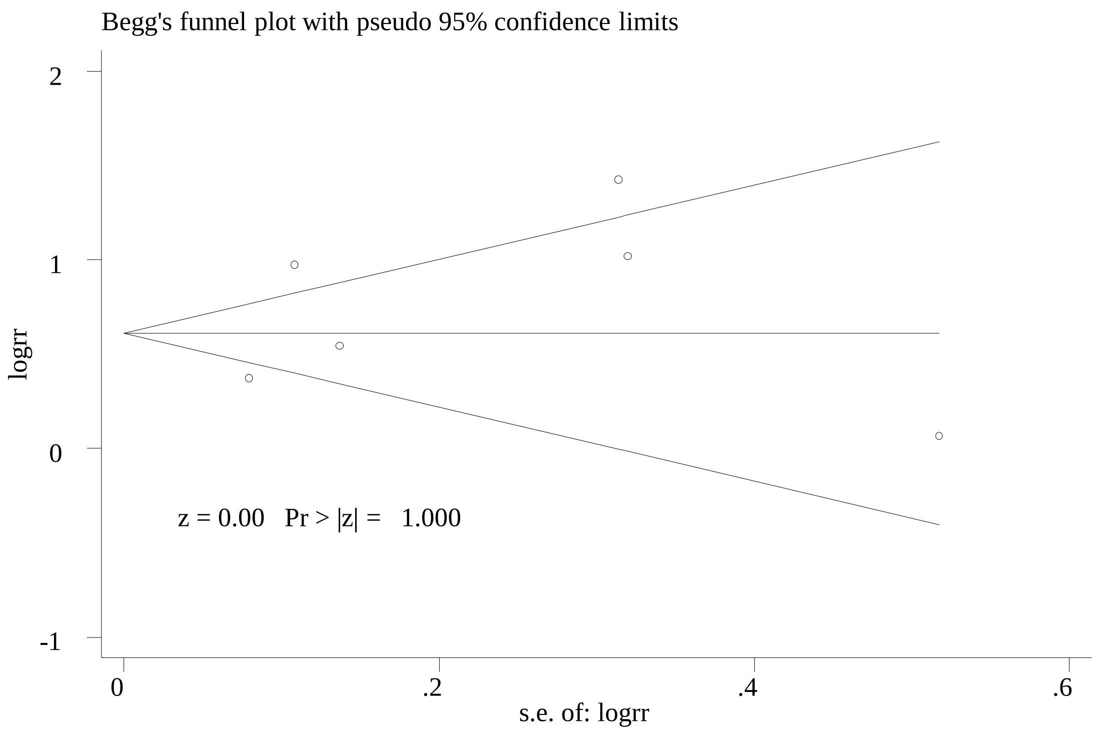

Supplement Figure 4. Begg‘s funnel plot of proportions of responders with normokalemia between the SZC and placebo group.

Table S1. Inherent risk of bias of included trials

Trial	Sequence generation	Allocation concealment	Blinding			Incomplete outcome data	Selective outcome reporting	Other source of bias
			participants	personnel	outcome assessors			
David 2015	UNCLEAR	UNCLEAR	LOW	LOW	LOW	LOW	UNCLEAR	UNCLEAR
DIALIZE 2019	LOW	LOW	LOW	LOW	LOW	LOW	LOW	UNCLEAR
ENERGIZE 2020	LOW	LOW	LOW	LOW	LOW	LOW	LOW	UNCLEAR
HARMONIZE2014	LOW	LOW	LOW	LOW	LOW	LOW	LOW	UNCLEAR
HARMONIZE 2015	LOW	LOW	LOW	LOW	LOW	LOW	LOW	UNCLEAR
HARMONIZE 2019	LOW	LOW	LOW	LOW	LOW	LOW	LOW	UNCLEAR
Kashihara2020	LOW	LOW	LOW	LOW	LOW	LOW	LOW	UNCLEAR
Stephen 2015	LOW	UNCLEAR	LOW	LOW	LOW	LOW	LOW	UNCLEAR

Assessment of risk bias according to the Cochrane collaboration tool, low risk of bias was represented as “LOW” and high risk of were “HIGH”.
