# Supplementary material for: Effects and Safety of a Novel Oral Potassium-Lowering Drug-Sodium Zirconium Cyclosilicate for the Treatment of Hyperkalemia: a Systematic Review and Meta-Analysis
Source: Cardiovasc Drugs Ther. 2021 Jan 18;35(5):1057–66. doi: 10.1007/s10557-020-07134-2 (PMC8452568; doi:10.1007/s10557-020-07134-2)
Supplement: Supplementary file 2 — (DOCX 30 kb) [file 10557_2020_7134_MOESM2_ESM.docx]

PROTOCOL First drafted in September 2020

Updated in September 2020

**Effects of A Novel Oral Potassium-lowering Drug-Sodium Zirconium Cyclosilicate for the Treatment of Hyperkalemia: a systematic review and meta-analysis**

Yaru Zhang^1^ Saiqiu Liu ^1^ Youxia Liu^2^ Junying Xu^1^ Lihong Long^1^ Junya Jia^2^ Shan Lin^2^ Tiekun Yan^2^

**Author affiliations:** ^1^Department of Nephrology, Hunan Second People's Hospital, Hunan, China; ^2^ Department of Nephrology, General Hospital of Tianjin Medical University, Tianjin, China.

**Objectives and aims:**

We undertook a systematic review and meta-analysis to evaluate the effect of a novel oral potassium-lowering drug-sodium zirconium cyclosilicate for the treatment of hyperkalemia.

**Background**

Hyperkalemia is a common electrolyte disorder that can cause potentially life-threatening arrhythmias [1]. Hyperkalemia prevalence was about 7% [2] in a large healthcare system. Risks for hyperkalemia in chronic kidney disease (CKD, 22.8%), diabetes (10.8%), heart failure (HF, 13.4%) and use of renin-angiotensin-aldosterone system inhibitor (RAASi, 14.2%) were increased [3]. Prevalence increased in accordance with the severity of CKD [3]. The use of RAASi is the main treatment for CKD, which could reduce the risk of cardiovascular and renal adverse events and all-cause mortality [4, 5], however, their use is limited by some side effects, such as increased serum potassium (K), which is especially severe in patients with renal insufficiency [6]. It is worth noting that after the first hyperkalemia attack, 53.7% or 13.1% of patients experienced RAASi withdrawal or dose reduction, respectively [3]. Besides, 55.6 % of patients experiencing recurrence at 1 year for serum potassium (sK+) ≥5.1 mEq/l, 19.9% for sK+≥5.5 mEq/l, and 4.9% for ≥6.0 mEq/l [3].Therefore, it is imperative to strengthen the management of hyperkalemia, especially for patients with CKD and hyperkalemia.

Over the years, the treatment for hyperkalemia mainly included intravenous medications (intravenous insulin + glucose and diuretics), correction of acidosis, oral medication (such as diuretics, potassium-reducing resin) and so on [7]. Although intravenous insulin and glucose therapy can quickly reduce blood potassium levels by transferring potassium ions into cells, it does not change the total amount of potassium in the body, and the maintenance time is short [7, 8]. In addition, diuretics that promote the excretion of potassium ions by the kidneys are not selective for ions, and long-term use can cause renal impairment in patients [8]. Potassium-reducing resins that excrete potassium ions through the intestine also have low selectivity for potassium ions, and their therapeutic effects and onset time have greater limitations [7].Sodium zirconium cyclosilicate (ZS-9) is a stable inorganic crystal, its structure highly matches the diameter of potassium ions [9, 10], and its binding force with "potassium" is 25 times than those of other cations [9, 10]. ZS-9 binds potassium ions in the entire digestive tract, which promotes the transfer of blood potassium to the intestine and excretes the body, thereby achieving rapid potassium reduction and long-term potassium control [10, 11]. The data showed that a significant drop in blood potassium levels was observed in 1 hour after receiving ZS-9 treatment [12], and 98% of patients returned to normal range within 48 hours; nearly 99% of patients had serum potassium levels <5.5 mmol/L in one year [13]. To the best of our knowledge, we undertook this meta-analysis to summarize and evaluate the evidence surrounding ZS-9 for the first time.

**Research Plan:**

**A) Methods of the review**

The study will be conducted according to the PRISMA statement for the conduct of meta-analyses of intervention studies.

**B) Data sources:**

Relevant randomized controlled trials will be identified by computerized searches from the following data sources without language restriction: MEDLINE (from 1950 to Sep 2020), EMBASE (from 1970 to Sep 2020), and the Cochrane Library database (from 1950 to Sep 2020)

**C) Study selection:**

Types of studies:

Randomized controlled trials, studying the therapeutic effects of sodium zirconium cyclosilicate in patients with hyperkalemia

Types of participants:

Inclusion criteria:

i. Study population comprised participants aged 18 years old or older with hyperkalemia (defined as sK+ 5.1 mmol/L);

ii. Comparison of sodium zirconium cyclosilicate and placebo;

iii. Reported study outcomes included the change in serum potassium (sK+), proportions of responders (defined as patients with sK+ <6.0 mmol/L between 1 and 4 hours, and <5.0 mmol/L at 4 hours, and not requiring additional therapy for hyperkalemia, and according to the definition criteria of the included studies), patient with normal serum kalaemia (defined as patients with sK+ <5.1 mmol/L, or according to the definition criteria of the included studies) between the ZS-9 and placebo group at the end of study, and/or safety data of drug-related adverse events;

iv. Study design was RCT and sample size was larger than 20.

Exclusion criteria: We excluded studies without control group or lack of available data.

Type of intervention:

Use the sodium zirconium cyclosilicate.

**D) Type of outcome measures:**

**Search/MESH terms 
MEDLINE (OVID)**

1. sodium zirconium cyclosilicate

2. SZC

3. ZS-9 or ZS9

4. (sodium zirconium cyclosilicate or SZC or ZS-9 orZS9).

5. hyperkalemia

6. Randomized controlled trial

7. Controlled clinical trial

8. Randomized.tw.

9. Placebo.tw.

10. Randomly.tw.

11.Trial.tw.

12. 0r/ 6-11

13. 4 and 5 and 12

**EMBASE**

1. sodium zirconium cyclosilicate

2. SZC

3. ZS-9 or ZS9

4 .or/1-3

5. hyperkalemia

6. Randomized controlled trial

7. Controlled clinical trial

8. blind and method

9. randomized and controlled and trial

10. random and allocation

11. or/6-10

95. 4 and 5 and11

**COCHRANE CONTROLLED TRIALS**

1. hyperkalemia

2. sodium zirconium cyclosilicate

3. SZC

4. ZS-9 or ZS9

5. Or/2-5

6. Randomized controlled trial.pt.

7. Controlled clinical trial.pt

8. Randomized.tw.

9. Placebo.tw.

10. Randomly.tw.

11.Trial.tw.

12. (random$ adj5 trial$).tw.

13. (random$ adj5 allocation$).tw.

14. (Blind$ adj5 method$).tw.

15. or/6-14

16. 1 and 5 and 15

**Reference**

[1] Khanagavi J, Gupta T, Aronow WS, et al. Hyperkalemia among hospitalized patients and association between duration of hyperkalemia and outcomes. Arch Med Sci. 2014. 10(2): 251-7.

[2] Nilsson E, Gasparini A, Ärnlöv J, et al. Incidence and determinants of hyperkalemia and hypokalemia in a large healthcare system. Int J Cardiol. 2017. 245: 277-284.

[3] Kashihara N, Kohsaka S, Kanda E, Okami S, Yajima T. Hyperkalemia in Real-World Patients Under Continuous Medical Care in Japan. Kidney Int Rep. 2019. 4(9): 1248-1260.

[4] Zhang Y, He D, Zhang W, et al. ACE Inhibitor Benefit to Kidney and Cardiovascular Outcomes for Patients with Non-Dialysis Chronic Kidney Disease Stages 3-5: A Network Meta-Analysis of Randomised Clinical Trials. Drugs. 2020 .

[5] Hao G, Wang Z, Guo R, et al. Effects of ACEI/ARB in hypertensive patients with type 2 diabetes mellitus: a meta-analysis of randomized controlled studies. BMC Cardiovasc Disord. 2014. 14: 148.

[6] Santoro A, Mandreoli M. [Hyperkalemia as a limiting factor in the use of drugs that block the Renin Angiotensin Aldosterone System (RAAS)]. G Ital Nefrol. 2018 .

[7] Palmer BF, Clegg DJ. Diagnosis and treatment of hyperkalemia. Cleve Clin J Med. 2017. 84(12): 934-942.

[8] Cowan AC, Gharib EG, Weir MA. Advances in the management of hyperkalemia in chronic kidney disease. Curr Opin Nephrol Hypertens. 2017. 26(3): 235-239.

[9] 周庆飞. Sodium zirconium cyclosilicate(Lokelma). 中国药物化学杂志. 028(006): 515.

[10] Stavros F, Yang A, Leon A, Nuttall M, Rasmussen HS. Characterization of structure and function of ZS-9, a K+ selective ion trap. PLoS One. 2014. 9(12): e114686.

[11] Yang A, Leon A, Nuttall M, Low JJ, Rasmussen HS. IN VITRO ION EXCHANGE CAPACITY AND SELECTIVITY OF ZS-9, A NOVEL, SELECTIVE CATION TRAP FOR THE TREATMENT OF HYPERKALEMIA. Am J Kidney Dis. 2014. 63(5): B115.

[12] Packham DK, Rasmussen HS, Lavin PT, et al. Sodium zirconium cyclosilicate in hyperkalemia. N Engl J Med. 2015. 372(3): 222-31.

[13] Spinowitz BS, Fishbane S, Pergola PE, et al. Sodium Zirconium Cyclosilicate among Individuals with Hyperkalemia: A 12-Month Phase 3 Study. Clin J Am Soc Nephrol. 2019. 14(6): 798-809.
